# Supplementary material for: Placental lipid handling, growth and inflammatory pathways are modified by a maternal Mediterranean diet
Source: Sci Rep. 2026 Jul 28;16:21820. doi: 10.1038/s41598-026-60877-0 (PMC13415775; doi:10.1038/s41598-026-60877-0)
Supplement: Supplementary file 2 — Supplementary Material 2 [file 41598_2026_60877_MOESM2_ESM.docx]

**Table S1.** List of primers used for qPCR.

|  | Forward Primer (5' to 3') | Reverse Primer (5' to 3') |
| --- | --- | --- |
| *SLC3A2* | GGAGTTTTGGCTGCAAGCTG | CCGCAATCAAGAGCCTGTCT |
| *SLC2A1* | TCCTCATCGCCCAGGTGTTC | CTCCTCGTTGCGGTTGATGA |
| *SLC2A8* | ATGTGCTTCATGCCCGAGACC | TGGATGACACCCACGACGA |
| *RAGE* | CCTGGAAGGAAGCAGGATGG | TCCGGCCTGTGTTCAGTTTC |
| *SOCS3* | CATCTCTGTCGGAAGACCGTCA | GCATCGTACTGGTCCAGGAACT |
| *PPARG* | CCAGAAGCCTGCATTTCTGC | GTGTCAACCATGGTCATTTCGTT |
| *ALOX15* | TGTGAAAGACGACCCAGAGC | AGAGACAGGAAACCCTCGGT |
| *PTGS2* | GCAAATTGCTGGCAGGGTTG | GCTCTGGTCAATGGAAGCCT |
| *GHR* | GAACCCGCGCTCTCTGAT | ATAGCTGCTGTGGCCTCACT |
| *ACADM* | GGGTTCGGGCGATGCTG | CTGCTGTTCGGTGAACTCAAA |
| *ACADL* | TCAGCTGATCGTCCTCCCTC | GAATGAGAACATCGCGCGGC |
| *ACADVL* | CCGGAGAGATTCGGAGATGC | TGAGCCGCGAGCTTCC |
| *FASN* | GCAAGCTGAAGGACCTGTCT | AATCTGGGTTGATGCCTCCG |
| *APOE* | GGGTCGCTTTTGGGATTACCTG | CAACTCCTTCATGGTCTCGTCC |
| *GAPDH* | AGCCACATCGCTCAGACAC | GCCCAATACGACCAAATCC |
| *HPRT* | ATCAGACTGAAGAGCTATTGTAATGACCA | TGGCTTATATCCAACACTTCGTG |

**Table S2.** List of antibodies used for western blotting.

| **Protein** | **Company, Catalogue #** | **Dilution** |
| --- | --- | --- |
| **Total p38 MAPK** | Cell signalling 8690 | 1:1000 |
| **Phosphorylated p38 MAPK** | Cell signalling 4511 | 1:1000 |
| **Phosphorylated AKT (Ser473)** | Cell signalling 9271 | 1:1000 |
| **Total AKT** | Cell signalling 9272 | 1:1000 |
| **Raptor** | Cell signalling 2280 | 1:1000 |
| **G-beta-L** | Cell signalling 3274 | 1:1000 |
| **PI3K-p85** | Milipore, 06-195 | 1:5000 |
| **PPAR gamma** | Santa Cruz-7273 | 1:200 |
| **MMP3** | Cell signalling 14351 | 1:1000 |
| **PAI1** | Cell signalling 49536 | 1:1000 |
