# Supplementary figures and images for: Placental lipid handling, growth and inflammatory pathways are modified by a maternal Mediterranean diet
Source: Sci Rep. 2026 Jul 28;16:21820. doi: 10.1038/s41598-026-60877-0 (PMC13415775; doi:10.1038/s41598-026-60877-0)

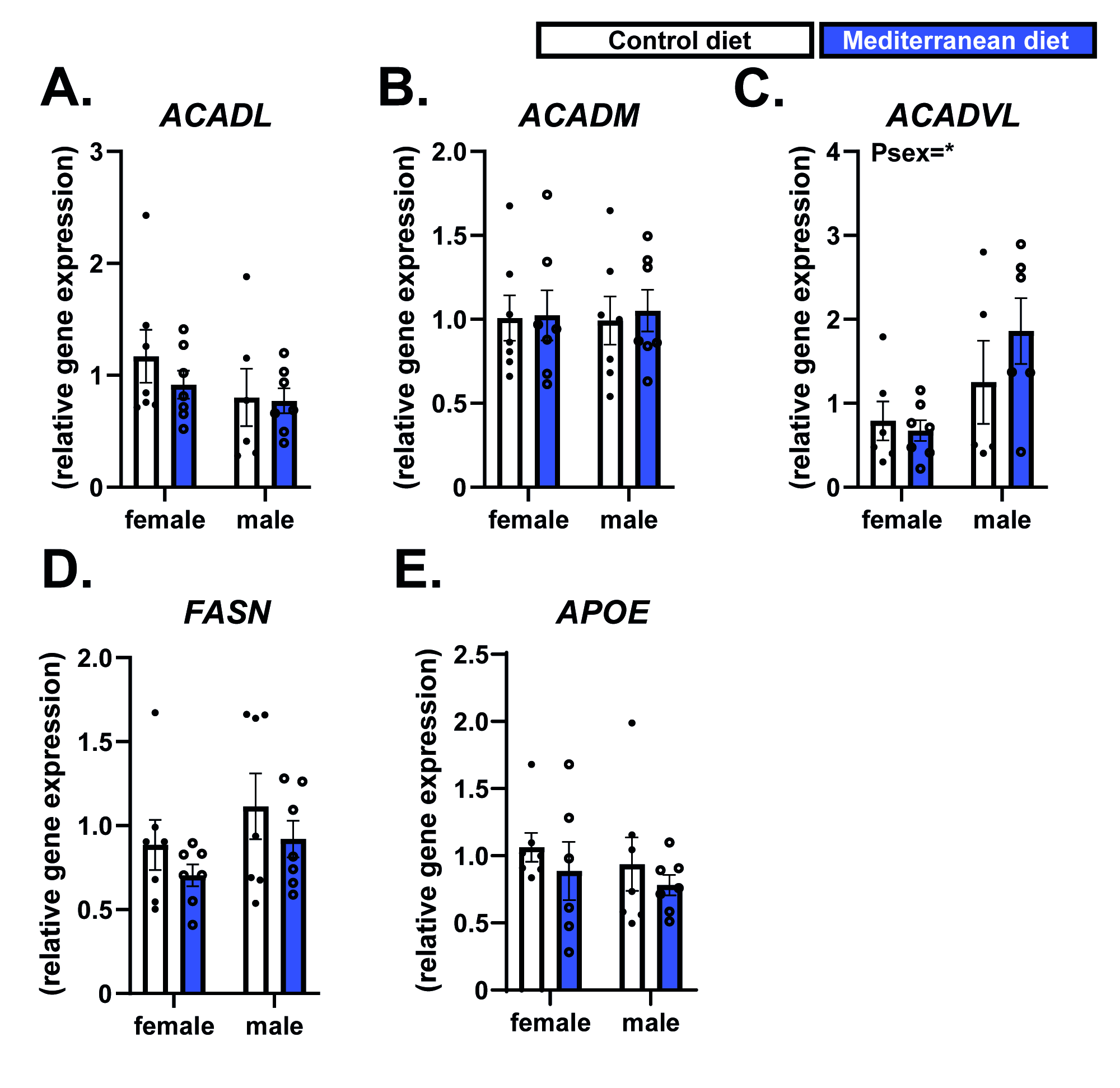

Supplement: Supplementary file 3 — Supplementary Material 3 [file 41598_2026_60877_MOESM3_ESM.tif]

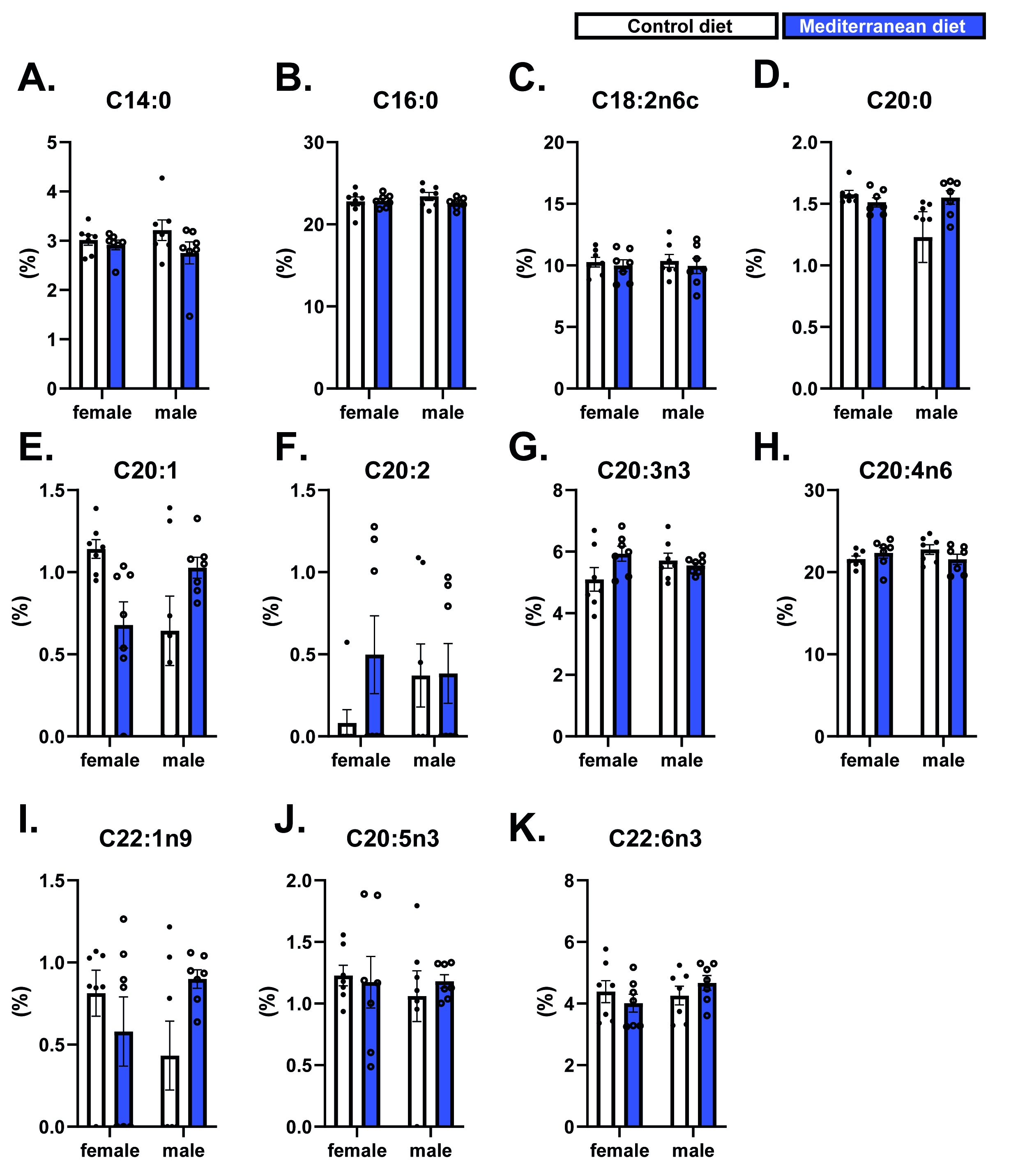

Supplement: Supplementary file 4 — Supplementary Material 4 [file 41598_2026_60877_MOESM4_ESM.tif]
