# Supplementary material for: Placental lipid handling, growth and inflammatory pathways are modified by a maternal Mediterranean diet
Source: Sci Rep. 2026 Jul 28;16:21820. doi: 10.1038/s41598-026-60877-0 (PMC13415775; doi:10.1038/s41598-026-60877-0)
Supplement: Supplementary file 1 — Supplementary Material 1 [file 41598_2026_60877_MOESM1_ESM.pdf]

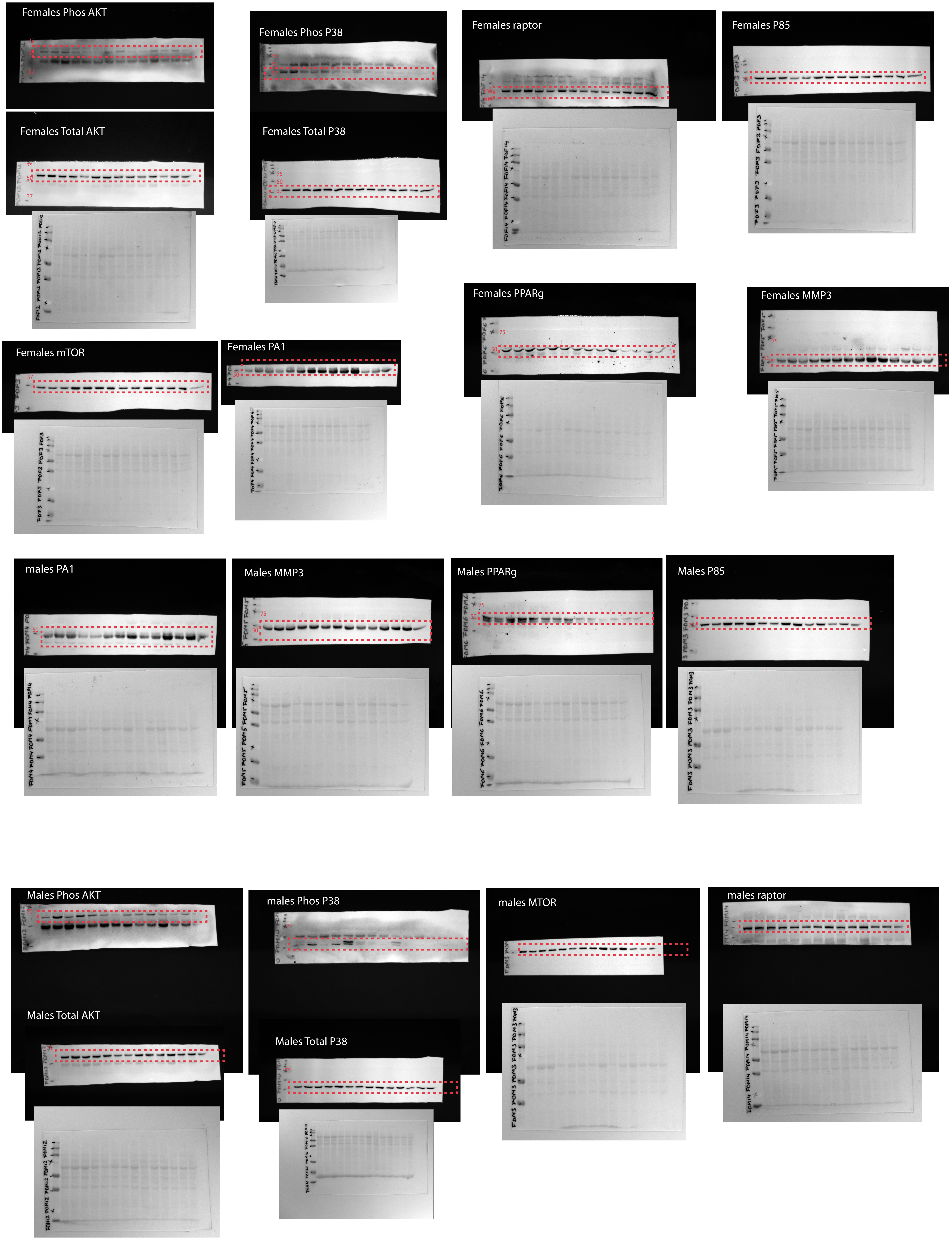
Females Phos AKT
Females Phos P38
Females raptor
Females P85
Females Total AKT
Females Total P38
Females PPARg
Females MMP3
Females mTOR
Females PA1
males PA1
Males MMP3
Males PPARg
Males P85
Males Phos AKT
males Phos P38
males MTOR
males raptor
Males Total AKT
Males Total P38
